# Allogeneic adipose tissue‐derived stem cells ELIXCYTE^®^ in chronic kidney disease: A phase I study assessing safety and clinical feasibility

**DOI:** 10.1111/jcmm.17310

**Published:** 2022-04-12

**Authors:** Cai‐Mei Zheng, I‐Jen Chiu, Yu‐Wei Chen, Yung‐Ho Hsu, Lie‐Yee Hung, Mei‐Yi Wu, Yuh‐Feng Lin, Chia‐Te Liao, Yi‐Pei Hung, Chia‐Chu Tsai, Yih‐Giun Cherng, Mai‐Szu Wu

**Affiliations:** ^1^ Division of Nephrology Department of Internal Medicine Taipei Medical University‐Shuang Ho Hospital New Taipei City Taiwan; ^2^ Division of Nephrology Department of Internal Medicine School of Medicine College of Medicine Taipei Medical University Taipei Taiwan; ^3^ Taipei Medical University‐Research Center of Urology and Kidney (TMU‐RCUK) School of Medicine College of Medicine Taipei Medical University Taipei Taiwan; ^4^ College of Medicine Graduate Institute of Clinical Medicine Taipei Medical University Taipei Taiwan; ^5^ Division of Nephrology Department of Internal Medicine Hsin Kuo Min Hospital Taipei Medical University Taoyuan City Taiwan; ^6^ College of Public Health Institute of Epidemiology and Preventive Medicine National Taiwan University Taipei Taiwan; ^7^ UnicoCell BioMed Co. Ltd. Taipei Taiwan; ^8^ Department of Anesthesiology Shuang Ho Hospital Taipei Medical University New Taipei City Taiwan; ^9^ Department of Anesthesiology School of Medicine College of Medicine Taipei Medical University Taipei Taiwan

**Keywords:** allogeneic adipose tissue‐derived stem cells, chronic kidney disease, estimated glomerular filtration rate

## Abstract

The purpose of this phase I clinical trial is to assess the safety and tolerability of allogeneic adipose tissue‐derived stem cells (ADSCs) among chronic kidney disease (CKD) patients. 12 eligible CKD patients with an estimated glomerular filtration rate (eGFR) of 15–44 ml/min/1.73 m^2^ received one dose of intravenous allogeneic ADSCs (ELIXCYTE^®^), as 3 groups: 3 low dose (6.4 × 10^7^ cells in total of 8 ml), 3 middle dose (19.2 × 10^7^ cells in total of 24 ml) and 6 high dose (32.0 × 10^7^ cells in total of 40 ml) of ELIXCYTE^®^ and evaluated after 48 weeks. Primary endpoint was the safety profiles in terms of incidence of adverse events (AEs) and serious adverse event (SAE). Two subjects in high dose group experienced a total of 2 treatment‐related AEs which are Grade 1 slow speech and Grade 1 bradyphrenia after the infusion. One subject in middle dose group experienced an SAE unlikely related to treatment, grade 2 proteinuria. No fatal AE was reported in this study. An increase in eGFR was observed in 7 out of 12 subjects (58%) at Week 24 and in 6 of 12 subjects (50%) by Week 48. By Week 24, an increase in eGFR by more than 20% among all CKD patients with baseline eGFR ≧ 30 ml/min/1.73 m^2^ as compared to only 2 subjects in baseline eGFR < 30 ml/min/1.73 m^2^ group. No significant reduction in proteinuria was noted among all subjects. This phase I trial demonstrated single‐dose intravenous ELIXCYTE was well tolerated in moderate‐to‐severe CKD patients and its preliminary efficacy warrants future studies.

## 1 INTRODUCTION

Chronic kidney disease (CKD) is a globally rising non‐communicable disease with estimated global incidence of 11%–13%^1^ and is important for its progressive nature which finally ensuing in end‐stage kidney disease (ESKD).^2^ Since kidney plays an important local role in several body homeostatic mechanisms including electrolyte balance, blood pressure control, mineral metabolism and haematopoiesis, the whole disease spectrum from early to late CKD associate with significant morbidity and mortality. The initiation of renal replacement therapy and/or transplantation in ESKD patients further inherent a huge burden on healthcare systems.^3^ This pursues the idea of novel therapeutic interventions to retard the CKD progression to ESRD.^4^, ^5^, ^6^ Regenerative cell‐based therapy is one of the extensively studied novel treatments in CKD.

Chronic kidney disease is characterized by reduced kidney regenerative capacity in both renal repair and regrowth of partial or whole nephrons. In recent years, multipotent mesenchymal stem cells (MSCs) are of great interest for cell‐based therapy related to their novel role in tissue regeneration and repair.^7^ Various CKD models including diabetic nephropathy and allograft nephropathy revealed the ability of MSCs to preserve renal structure and improve renal function.^8^, ^9^, ^10^, ^11^, ^12^, ^13^ Among MSCs, adipose tissue‐derived stem cells (ADSCs) have better anti‐inflammatory and immunomodulating actions than bone marrow derived MSCs (BDMSCs).^14^ Additionally, ADSCs are more feasible, high yield and lower immunogenicity than BDMCs. Zhu XY et al. found that intrarenal administration of ADSCs in animal renovascular disease improve the renal function.^15^, ^16^ Further, other studies also proved that intrarenal MSC delivery and revascularization improved hypoxia, inflammation, oxidative stress and subsequent renal fibrosis.^17^, ^18^ On the contrary, ADSCs have been proved to reduce the severity of ischaemic‐reperfusion injury (IRI) and prevent renal fibrosis through inhibition of oxidative stress and inflammation.^14^, ^19^

Although several findings confirmed the beneficial effects of MSCs in these preclinical models of CKD, the diversity in CKD models, cell types and doses, administration route and different renal outcome parameters result in difficult judgement and further use in clinical settings. Few clinical trials have been investigated for the safety, dose and therapeutic potential of ADSCs, especially in different CKD patients. In this phase I clinical trial, we used ELIXCYTE^®^, an investigational product composed of allogeneic human adipose tissue‐derived stem cells (ADSCs) in low, middle and high dose to evaluate the safety and side effects among CKD patients with various underlying aetiologies.

## 2 METHODS

This single‐centre, dose‐escalation and open‐labelled phase I clinical trial in Taipei Medical University‐Shuang Ho Hospital enrolled a total of 12 adult patients with CKD from April 2018 to October 2019; each subject signed an informed consent before participating in the study. The study was registered on clinicaltrail.gov (NCT02933827) and was approved by the Taipei Medical University‐Joint Institutional Review Board (N201710032) adheres to the tenets of the Declaration of Helsinki.

### 2.1 Patients, inclusion/exclusion criteria

Eligible participants included patients aged 20–80 years with CKD stages 3b to 4, estimated glomerular filtration rate (eGFR) 15–44 ml/min/1.73 m^2^ (ml/min) (inclusive) using the Modification of Diet in Kidney Disease (MDRD) formula. We excluded those with hypersensitivity to any component used in the study, inadequate haematologic and hepatic function, uncontrolled diabetes mellitus (DM) with glycated haemoglobin A1c (HbA1c) >8.0%, human immunodeficiency virus (HIV) infection or any type of hepatitis, autoimmune diseases, cystic kidney disease or requiring any form of dialysis. Patients already participated in any other interventional study within 4 weeks of entering the study were also excluded (Table 2). Amphotericin B, vancomycin and amikacin would be prohibited from patients. They could receive routine medications/treatments for other indications as judged by study investigators.

### 2.2 Treatment protocol

Firstly, three CKD patients were treated with low dose ELIXCYTE^®^ (ADSCs 6.4 × 10^7^ cells) injection and would be observed for 14 days. If there were no safety concern after reviewed by Data and Safety Monitoring Board (DSMB), second and third three CKD patients would be given moderate dose ELIXCYTE^®^ (ADSCs 19.2 × 10^7^ cells) and high dose ELIXCYTE^®^ (ADSCs 32.0 × 10^7^ cells) sequentially after safety assessments based on DSMB recommendation. Each study participant would sign the informed consent form after 14 days counted from the day that former patient receiving the administration of the investigational product. Due to development of suspected drug administration related adverse event, we recruited 3 more subjects in high dose (ADSCs 32.0 × 10^7^ cells) group as DSMB suggestion. Finally, a total of 12 subjects, 3, 3 and 6 subjects, were treated with low, moderate and high dose ELIXCYTE^®^ separately in this study. One dose of intravenous administration was given to each patient, who were followed up regularly for a total of 48 weeks. Changes in vital signs, physical and laboratory examination from baseline to post treatment visits were closely monitored. The adverse events (AEs) and serious adverse events (SAEs) and eGFR and urine total protein changes from baseline were evaluated at 0, 2, 4, 8, 12, 24, 36 and 48 weeks after administration. All adverse events occurring after MSC infusion were actively monitored. Any event either related to the MSC treatment or not was judged by investigating physicians and reported as serious adverse effects or adverse effects. This study was planned and conducted under the rules of good clinical practice (GCP), including the design of the clinical protocol and other process documents, as well as the archiving of essential documents.

### 2.3 Preparation of allogeneic human adipose tissue‐derived stem cell product ELIXCYTE^®^

The donor was recruited under the supervision of an IRB in National Taiwan University Hospital and was screened and tested in compliance with eligibility determination guidance issued by Taiwan Food and Drug Administration (TFDA). The adipose tissue was collected from eligible donor via ultrasonic‐assisted liposuction and was transferred to UnicoCell BioMed Company under hypothermal condition within 6 h. The stromal vascular fraction (SVF) was isolated by digesting the adipose tissue with type I collagenase, centrifuged and cryopreserved in liquid nitrogen tank for quarantine release. Following SVF met the release specification, SVF was revived, inoculated in T‐flask and cultured at 37°C in a humidified carbon dioxide (CO_2_) incubator for primary culture. After approximately 7 days in culture, the adherent and highly proliferated cells termed ADSCs were detached and propagated to the predefined population doubling level (PDL). The ADSCs were then harvested and formulated at a density of 8 × 10^6^ cells/ml with cryoprotectant CryoStor^®^ CS10 (BioLife Solutions) containing 10% of DMSO (v/v). A gently pipetting was performed to obtain a single‐cell suspension which was evenly filled to closure cryotubes. The cryotubes were cooled by a control rate freezer and temporarily stored in a vapour phase liquid nitrogen tank for quarantine release. Upon the criteria were met, the medicinal product named ELIXCYTE^®^ was transferred to qualified area for long term storage. Release testing of ELIXCYTE^®^ for microbiological evaluation (including mycoplasma, sterility and endotoxin tests) were employed to ensure safety. The product characteristics such as identity (including MSC markers, viable cell count and cell viability), product related impurities (immunophenotyping for CD34/CD45/CD11b/CD19/HLA‐DR) and tri‐lineage differentiation property were also assessed.

### 2.4 Statistical analysis

The variables were reported as mean ± standard deviation or median (interquartile). The differences in changes between the groups were analysed by the unpaired *t*‐test. The differences in changes within groups were analysed by the paired *t*‐test. For non‐normal distributed variable, Mann–Whitney *U*‐test was used to analyse the difference between the two groups. The statistical analyses were performed by using the SPSS version 16.0 statistical software program.

## 3 RESULTS

### 3.1 Disposition of subjects

**FIGURE 1:**
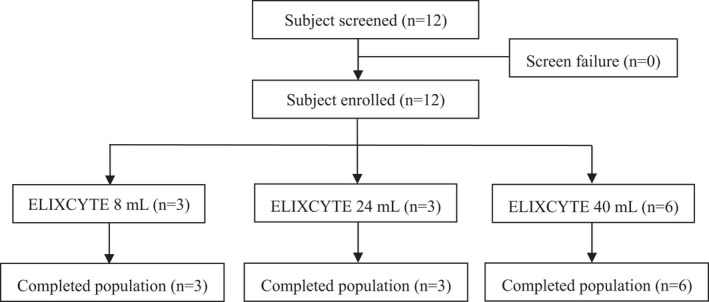
Subject disposition

Disposition of subjects participating in this clinical study is presented in Table 1. Enrolment status by study site is also summarized in Figure 1. Twelve subjects were screened for the study at Taipei Medical University Shuang Ho Hospital, and all subjects were eligible to be enrolled to the study. Patients were administered with one of three dose levels of investigational product based on the enrolled order. All the eligible subjects completed the study and were included in efficacy and safety analyses.

**TABLE 1:** Disposition of subjects

Characteristics	ELIXCYTE 8 ml	ELIXCYTE 24 ml	ELIXCYTE 40 ml	All Dose level
Screened subjects				12
Enrolled subjects	3	3	6	12
Analysis population				
Treated subjects	3 (100%)	3 (100%)	6 (100%)	12 (100%)
Completed visit 9 (Final, Day 337 [Week48])	3 (100%)	3 (100%)	6 (100%)	12 (100%)
Early termination	0 (0.0%)	0 (0.0%)	0 (0.0%)	0 (0.0%)

Note

All percentages have the denominator as the total number of enrolled subjects.

### 3.2 Demography

The demographic data of all treated subjects (*n* = 12) are summarized in Table 2. Result showed that mean (±SD) age of all enrolled subjects was 53.4 ± 10.85. Their mean (±SD) BMI was 25.0 ± 4.61. Ten males and 2 females were enrolled in the study.

**TABLE 2:** Patient characteristics and demographic data at the time of enrolment

Patient number	02‐001	02‐002	02‐003	02‐004	02‐005	02‐006	02‐007	02‐008	02‐009	02‐010	02‐011	02‐012
Sex (M/F)	M	F	M	M	M	M	M	M	F	M	M	M
Age (years)	56	36	71	54	62	62	53	54	40	40	65	47
BMI (kg/m^2^)	25.9	34	24.4	24.45	23.07	28.38	23.77	22.86	17.68	24.19	31.39	19.49
Aetiology of CKD	CIN	CIN	CIN	DM	HTN	HTN	DM	HTN	CIN	HTN	DM	CIN
eGFR (ml/min/1.73 m^2^)	21.94	26.58	35.28	26.53	25.04	21.02	23.47	16.15	37.40	43.52	17.83	24.33
Serum Cr (mg/dl)	3.15	2.23	2.0	2.68	2.75	3.2	3.00	4.12	1.62	1.84	3.66	2.96
Urine Cr (mg/dl)	140.5	148.1	184.8	145.0	148.5	88.0	87.8	113.5	120.6	51.8	117.8	44.4
Urine Protein (mg/dl)	109.4	95.3	15.7	562.1	69.1	107.3	44.5	41.0	14.0	<6.0	154.5	16.7
Urine Protein/Creatine	0.78	0.64	0.08	3.88	0.47	1.22	0.51	0.36	0.12	<0.12	1.31	0.38

Abbreviations: BMI, body mass index; CIN, chronic interstitial nephritis; CKD, chronic kidney disease; Cr, creatinine; DM, diabetes mellitus; eGFR, estimated glomerular filtration rate; F, female; HTN, hypertension; M, male.

### 3.3 Adverse events

Incidence of AEs and SAEs are the primary endpoint in this phaseⅠstudy. Forty‐one of AEs were reported by 11 subjects (11 out of 12 treated subjects, 91.7%) who received the investigational product in this study. Most AEs were Grade 1 to Grade 2 in severity, only 1 subject (1 out of 12, 8.3%) who received middle dose experienced an AE with Grade 3 severity. Study treatment‐related AEs occurred to 2 subjects in high dose level. There was 1 serious adverse event reported in middle dose level and no fatal AE. A summary of adverse events for safety analysis in this study is shown in Table 3.

**TABLE 3:** Summary of adverse events

Characteristics	ELIXCYTE 8 ml	ELIXCYTE 24 ml	ELIXCYTE 40 ml	All Dose level
No. of AEs	11	8	22	41
No. of subjects	3	3	6	12
No. of subjects with AEs	3 (100.0%)	3 (100.0%)	5 (83.3%)	11 (91.7%)
No. of subjects with Grade 3/4/5 AEs	0 (0.0%)	0 (0.0%)	0 (0.0%)	0 (0.0%)
No. of subjects with study treatment‐related AEs	0 (0.0%)	0 (0.0%)	2 (33.3%)	2 (16.7%)
No. of subjects with withdrawn of study treatment due to AEs	0 (0.0%)	0 (0.0%)	0 (0.0%)	0 (0.0%)
No. of subjects with SAEs	0 (0.0%)	1 (33.3%)	0 (0.0%)	1 (8.3%)
No. of subjects with fatal AEs	0 (0.0%)	0 (0.0%)	0 (0.0%)	0 (0.0%)

Abbreviations: AE, adverse event; No., number; SAE, serious adverse event.

All AEs were coded using medical dictionary for regulatory activities (MedDRA) version 22.1 and summarized by system organ class (SOC) and preferred term (PT) with number of subjects and incidence (%) of events in each treatment group. 11 out of 12 subjects experienced at least one AE during the study. Overall, 41 AEs were recorded after the dose administration. Incidence of adverse events displayed by MedDRA SOC and PT are shown in Table 4. The most common SOCs of AEs among 3 dose levels by incidence were infections and infestations (50%), gastrointestinal disorders (41.7%), connective tissue disorders (25.0%), psychiatric disorders (25.0%) and with others equal or less than 16.7%. In terms of PT, the most common AEs (>10%) in this study among three dose levels included constipation (16.7%), upper respiratory tract infection (16.7%), flank pain (16.7%) and insomnia (16.7%). The intensity, causality and other information of events are summarized using an event‐based approach in Table 5. Among the 41 AEs, one Grade 3 AE, acute pancreatitis (PT: pancreatitis acute), was observed in a patient receiving middle dose of ELIXCYTE. 2 AEs (4.9%), under the impression of slow speech (PT: slow speech) and slow response (PT: bradyphrenia), were observed in 2 patients receiving the high dose and assessed by the investigator as possibly related to study treatment.

**TABLE 4:** Incidence of adverse events (By PT and SOC)

Characteristics	ELIXCYTE 8 ml	ELIXCYTE 24 ml	ELIXCYTE 40 ml	All Dose level
No. of subjects	3	3	6	12
*At least one below*	3 (100.0%)	3 (100.0%)	5 (83.3%)	11 (91.7%)
Blood and lymphatic system disorders	1 (33.3%)	0 (0.0%)	0 (0.0%)	1 (8.3%)
Anaemia	1 (33.3%)	0 (0.0%)	0 (0.0%)	1 (8.3%)
Eye disorders	2 (66.7%)	0 (0.0%)	0 (0.0%)	2 (16.7%)
Cataract	1 (33.3%)	0 (0.0%)	0 (0.0%)	1 (8.3%)
Iridocyclitis	1 (33.3%)	0 (0.0%)	0 (0.0%)	1 (8.3%)
Gastrointestinal disorders	1 (33.3%)	2 (66.7%)	2 (33.3%)	5 (41.7%)
Abdominal pain upper	0 (0.0%)	1 (33.3%)	0 (0.0%)	1 (8.3%)
Constipation	1 (33.3%)	0 (0.0%)	1 (16.7%)	2 (16.7%)
Diarrhoea	0 (0.0%)	0 (0.0%)	1 (16.7%)	1 (8.3%)
Gingival bleeding	1 (33.3%)	0 (0.0%)	0 (0.0%)	1 (8.3%)
Pancreatitis acute	0 (0.0%)	1 (33.3%)	0 (0.0%)	1 (8.3%)
General disorders and administration site conditions	0 (0.0%)	1 (33.3%)	0 (0.0%)	1 (8.3%)
Chest discomfort	0 (0.0%)	1 (33.3%)	0 (0.0%)	1 (8.3%)
Infections and infestations	1 (33.3%)	3 (100.0%)	2 (33.3%)	6 (50.0%)
Bronchitis	0 (0.0%)	1 (33.3%)	0 (0.0%)	1 (8.3%)
Herpes zoster	0 (0.0%)	1 (33.3%)	0 (0.0%)	1 (8.3%)
Periodontitis	0 (0.0%)	0 (0.0%)	1 (16.7%)	1 (8.3%)
Rash pustular	1 (33.3%)	0 (0.0%)	0 (0.0%)	1 (8.3%)
Upper respiratory tract infection	0 (0.0%)	1 (33.3%)	1 (16.7%)	2 (16.7%)
Injury, poisoning and procedural complications	0 (0.0%)	0 (0.0%)	1 (16.7%)	1 (8.3%)
Contusion	0 (0.0%)	0 (0.0%)	1 (16.7%)	1 (8.3%)
Foot fracture	0 (0.0%)	0 (0.0%)	1 (16.7%)	1 (8.3%)
Forearm fracture	0 (0.0%)	0 (0.0%)	1 (16.7%)	1 (8.3%)
Foreign body in gastrointestinal tract	0 (0.0%)	0 (0.0%)	1 (16.7%)	1 (8.3%)
Investigations	1 (33.3%)	0 (0.0%)	1 (16.7%)	2 (16.7%)
Hepatic enzyme increased	0 (0.0%)	0 (0.0%)	1 (16.7%)	1 (8.3%)
Low density lipoprotein increased	1 (33.3%)	0 (0.0%)	0 (0.0%)	1 (8.3%)
Metabolism and nutrition disorders	0 (0.0%)	0 (0.0%)	1 (16.7%)	1 (8.3%)
Hyperkalaemia	0 (0.0%)	0 (0.0%)	1 (16.7%)	1 (8.3%)
Hyperuricaemia	0 (0.0%)	0 (0.0%)	1 (16.7%)	1 (8.3%)
Hypokalaemia	0 (0.0%)	0 (0.0%)	1 (16.7%)	1 (8.3%)
Musculoskeletal and connective tissue disorders	1 (33.3%)	0 (0.0%)	2 (33.3%)	3 (25.0%)
Flank pain	1 (33.3%)	0 (0.0%)	1 (16.7%)	2 (16.7%)
Musculoskeletal pain	0 (0.0%)	0 (0.0%)	1 (16.7%)	1 (8.3%)
Nervous system disorders	1 (33.3%)	0 (0.0%)	1 (16.7%)	2 (16.7%)
Dizziness	1 (33.3%)	0 (0.0%)	0 (0.0%)	1 (8.3%)
Slow speech	0 (0.0%)	0 (0.0%)	1 (16.7%)	1 (8.3%)
Psychiatric disorders	0 (0.0%)	1 (33.3%)	2 (33.3%)	3 (25.0%)
Bradyphrenia	0 (0.0%)	0 (0.0%)	1 (16.7%)	1 (8.3%)
Insomnia	0 (0.0%)	1 (33.3%)	1 (16.7%)	2 (16.7%)
Renal and urinary disorders	0 (0.0%)	1 (33.3%)	1 (16.7%)	2 (16.7%)
Dysuria	0 (0.0%)	0 (0.0%)	1 (16.7%)	1 (8.3%)
Proteinuria	0 (0.0%)	1 (33.3%)	0 (0.0%)	1 (8.3%)
Respiratory, thoracic and mediastinal disorders	1 (33.3%)	0 (0.0%)	1 (16.7%)	2 (16.7%)
Cough	0 (0.0%)	0 (0.0%)	1 (16.7%)	1 (8.3%)
Productive cough	1 (33.3%)	0 (0.0%)	0 (0.0%)	1 (8.3%)
Skin and subcutaneous tissue disorders	1 (33.3%)	0 (0.0%)	0 (0.0%)	1 (8.3%)
Pruritus	1 (33.3%)	0 (0.0%)	0 (0.0%)	1 (8.3%)

**TABLE 5:** Summary of adverse events (Event‐based)

Characteristics	ELIXCYTE 8 ml	ELIXCYTE 24 ml	ELIXCYTE 40 ml	All Dose level
Number of AEs	11	8	22	41
Event by intensity				
Grade 1	8 (72.7%)	4 (50.0%)	21 (95.5%)	33 (80.5%)
Grade 2	3 (27.3%)	3 (37.5%)	1 (4.5%)	7 (17.1%)
Grade 3	0 (0.0%)	1 (12.5%)	0 (0.0%)	1 (2.4%)
Grade 4	0 (0.0%)	0 (0.0%)	0 (0.0%)	0 (0.0%)
Grade 5	0 (0.0%)	0 (0.0%)	0 (0.0%)	0 (0.0%)
Unknown	0 (0.0%)	0 (0.0%)	0 (0.0%)	0 (0.0%)
Event by relationship to study treatment				
Definitely related	0 (0.0%)	0 (0.0%)	0 (0.0%)	0 (0.0%)
Probably related	0 (0.0%)	0 (0.0%)	0 (0.0%)	0 (0.0%)
Possibly related	0 (0.0%)	0 (0.0%)	2 (9.1%)	2 (4.9%)
Unlikely	0 (0.0%)	2 (25.0%)	0 (0.0%)	2 (4.9%)
Not related	11 (100.0%)	6 (75.0%)	20 (90.9%)	37 (90.2%)
Unknown	0 (0.0%)	0 (0.0%)	0 (0.0%)	0 (0.0%)
Event by seriousness				
Serious	0 (0.0%)	1 (12.5%)	0 (0.0%)	1 (2.4%)
Non‐serious	11 (100.0%)	7 (87.5%)	22 (100.0%)	40 (97.6%)
Event by dose‐limiting toxicity				
Yes	0 (0.0%)	0 (0.0%)	1 (4.5%)	1 (2.4%)
No	11 (100.0%)	8 (100.0%)	21 (95.5%)	40 (97.6%)

### 3.4 Death, other serious adverse events and other significant adverse events

No death occurred during the conduct of this study. Of the 12 treated patients who completed 48 weeks' follow‐up, only one serious adverse event was reported in one subject (02‐004). The event was reported as proteinuria (SOC: renal and urinary disorders; PT: proteinuria) with Grade 2 in severity which resulted in hospitalization of the subject. The event of proteinuria had been confirmed as the symptom of poorly controlled DM and CKD. The causal relationship of the event to the study treatment was assessed as ‘unlikely’ by investigator.

### 3.5 Other significant adverse events—Dose‐limiting toxicities

Dose‐limiting toxicity (DLT) is defined as any toxicity observed during the first 14 days after administration which meets the predefined criteria as per protocol Section 4.4 and of which the causality to administration ELIXCYTE cannot be reasonably ruled out as judged by investigator. Of the 3 dose levels, no DLT was observed in patients received low and middle dose of ELIXCYTE. One DLT was observed in 1 of the 3 patients initially enrolled to the High dose of ELIXCYTE. Subject 02‐008 received a single dose of high dose ELIXCYTE (40 ml, ADSC 32.0 × 10^7^ cells) and complained about an adverse event ‘slow speech (PT: slow speech), grade 1’ immediately after dose administration. The AE was resolved on the next day and was considered as ‘possibly related’ to the study treatment and confirmed under the SOC of ‘Nervous system disorder’. A planned data safety monitoring board (DSMB) meeting was convened after 3 subjects received high dose ELIXCYTE and evaluated for at least 14 days. The board reviewed the post treatment safety profiles of the 3 subjects and suggested to expand the cohort sequentially in order to better understand the incidence of the identified DLT, and to continuously follow subjects' outcome. No other DLT was observed in the newly enrolled subjects in the high dose level. Board members of DSMB had agreed that all 3 dose levels were well tolerable to the intended population of the current protocol and approved to proceed to Phase II study for all 3 dose levels.

### 3.6 Change of estimated glomerular filtration rate

**FIGURE 2:**
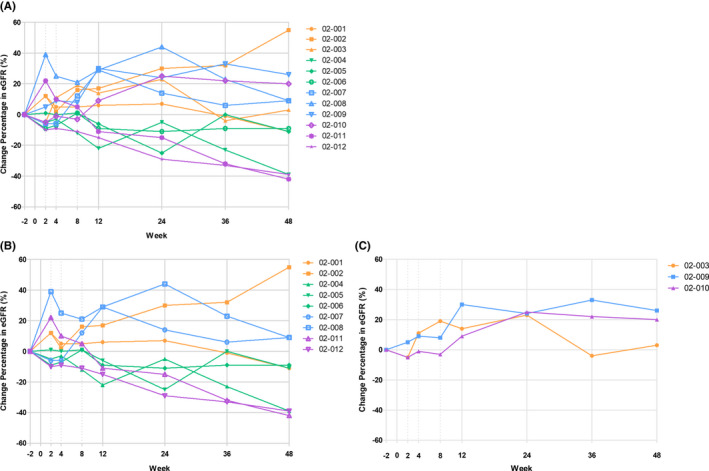
eGFR changes from baseline vs. visit over 48 weeks in (A) all treated subjects; (B) baseline eGFR <30 ml/min; and (C) baseline eGFR ≧30 ml/min. eGFR, estimated glomerular filtration rate

As for preliminary efficacy data, the change from baseline to Week 24 visit in estimated glomerular filtration rate was evaluated. The eGFR changes from baseline versus visit over 48 weeks in all treated subjects were shown in Figure 2A. An increase in eGFR was observed in 7 out of 12 subjects (58%) at Week 24 and in 6 of 12 subjects (50%) at Week 48. The increased percentages of eGFR at Week 24 by more than 20% were noted in all subjects with baseline eGFR ≧ 30 ml/min/1.73 m^2^ (Figure 2C). On the contrary, only 2 subjects with baseline eGFR < 30 ml/min/1.73 m^2^ acquired increased eGFR more than 20% by 24 weeks (Figure 2B). However, no significant reduction in proteinuria was noted in all subjects.

## 4 DISCUSSION

We described a phase I clinical trial on ELIXCYTE^®^, an allogeneic human adipose tissue‐derived stem cells (ADSCs) for improving kidney survival among chronic kidney disease (CKD) patients. Study subjects had estimated glomerular filtration rate (eGFR), assessed by the Modification of Diet in Kidney Disease, of 15–44 ml/min/1.73 m^2^. The underlying cause of CKD differs individually, and generally can be grouped into 5 chronic interstitial nephritis (CIN), 3 diabetes mellitus and 4 hypertensive nephrosclerosis. All patients took regular medications including renin‐angiotensin blockers before and during enrolment of the study. Generally, all our CKD patients well tolerated to three dose regimens of ELIXCYTE^®^. One dose‐limiting toxicity (DLT) was observed in high dose and the subject experienced grade 1 slow speech immediately after the administration, and the situation was resolved on the next day and afterwards. None of other subjects in the high dose cohort experienced DLT during the 14‐day observation period after infusion. All other reported SAE considered secondarily to underlying diseases.

The optimal dose and frequency of MSCs infusion in CKD patients are still unestablished. Intravenous administration of MSCs will result in a significant reduction in number of MSC arrived kidney. In this study, single‐dose intravenous infusion of ELIXCYTE at dose ranged from 6.4 × 10^7^ to 32.0 × 10^7^ ADSC cells was well tolerated in patients with moderate‐to‐severe CKD. However, there is no difference in the preliminary observation of the change in eGFR among three doses cohorts. An increase in eGFR was observed in 7 out of 12 subjects (58%) at Week 24 and in 6 of 12 subjects (50%) at Week 48. In addition, a somehow stable or decrease in eGFR were observed in several subjects after Week 24. Therefore, whether there is a need for the second booster dose of treatment can be considered in future trials. In animal studies, weekly MSC administration significantly improve the remnant kidney function as compared with single dose.^20^ To summarize, the administration dose and frequency might be the critical factors determining renal function improvement during ADSC treatment in CKD patients.

Another interesting finding in our patients is that the increased percentages of eGFR at Week 24 by more than 20% were noted among those subjects with baseline eGFR ≧ 30 ml/min regardless of disease nature or injected ADSC dosage. Since CKD not only associates with loss of renal parenchyma, but also results in progressive renal fibrosis and capillary destruction,^21^ the earlier introduction of stem cell‐based therapy gains better results especially when structural changes are relatively mild.^22^ Moreover, various aetiologies of CKD result in damage to different cell types within the nephrons other than specified cell population, which restricted the effects of stem cell therapy. Packham DK et al. also revealed in a placebo‐controlled study of diabetic kidney disease patients that eGFR ≧ 30 ml/min/1.73 m^2^ group gained better outcomes from allogeneic bone marrow‐mesenchymal stem cells (BM‐MSCs) infusion.^23^ These findings confirmed that preserved residual renal status is important for stem cell therapy, and we proposed eGFR ≧ 30 ml/min/1.73 m^2^ group might be more suitable for such therapy, however, needed to be confirmed by larger clinical trials. We found no significant relation between dose of ADSCs and eGFR improvement; however, the results should be interpreted carefully and larger clinical trials are needed. A meta‐analysis by Papazova, D. A. et al.^9^ also found that there is no dose dependency in stem cell therapy, which is supposed by the fact that such therapy acts by switching on the endogenous repair not by supplying persistent source of exogenous cells. Indeed, ADSCs secrete cytokines and growth factors in a paracrine or autocrine manner to promote endogenous repair.^24^ Studies also demonstrated that ADSCs suppress T‐cell activation, induce reg‐T cells, inhibit apoptotic factors and further promote recovery of renal function.^25^, ^26^, ^27^, ^28^

This study aimed to evaluate safety and tolerability in CKD patients. Being a single‐centre dose‐escalation study, shorter observation period, limited number of subjects and variable study subject disposition became major limitations. We gave one intravenous infusion of ADSCs to patients; however, the frequency and dose of ADSCs in CKD are still controversial. Multiple administration compared with single dose might be beneficial for chronic conditions, since paracrine actions could reduce with time. Preclinical animal studies confirmed that weekly MSC administration significantly improve the kidney function than single dose.^20^ Thus, whether second booster dose is required is to be consider in future clinical trials.

In conclusion, we proved the safety and feasibility of allogeneic ADSCs in CKD patients. Whether underlying aetiology and stage of CKD, dose and frequency of ADSCs, and comorbid condition influence the efficacy of ADSCs in these patients is still needed to be considered by future larger clinical trials.

## CONFLICT OF INTEREST

No potential conflict of interest was reported by the authors.

## AUTHOR CONTRIBUTIONS

**Cai‐Mei Zheng:** Conceptualization (equal); Formal analysis (equal); Methodology (equal); Writing – original draft (equal); Writing – review & editing (equal). **I‐Jen Chiu:** Formal analysis (equal); Methodology (equal); Software (equal). **Yu‐Wei Chen:** Conceptualization (equal); Project administration (equal); Visualization (equal). **Yung‐Ho Hsu:** Data curation (equal); Investigation (equal); Methodology (equal). **Lie‐Yee Hung:** Methodology (equal); Software (equal); Validation (equal). **Mei‐Yi Wu:** Conceptualization (equal); Data curation (equal); Visualization (equal). **Yuh‐Feng Lin:** Formal analysis (equal); Visualization (equal); Writing – review & editing (equal). **Chia‐Te Liao:** Conceptualization (equal); Methodology (equal); Project administration (equal). **Yi‐Pei Hung:** Funding acquisition (equal); Resources (equal); Software (equal). **Chia‐Chu Tsai:** Funding acquisition (equal); Investigation (equal); Resources (equal). **Yih‐Giun Cherng:** Formal analysis (equal); Project administration (equal); Resources (equal); Supervision (equal); Writing – review & editing (equal). **Mai‐Szu Wu:** Funding acquisition (equal); Resources (equal); Validation (equal); Visualization (equal).

## Data Availability

Research data are not shared.
